# Assessment of genetically engineered *Trabulsiella odontotermitis* as a ‘Trojan Horse’ for paratransgenesis in termites

**DOI:** 10.1186/s12866-016-0822-4

**Published:** 2016-09-05

**Authors:** Chinmay Vijay Tikhe, Thomas M. Martin, Andréa Howells, Jennifer Delatte, Claudia Husseneder

**Affiliations:** Department of Entomology, Louisiana State University Agricultural Center, Baton Rouge, LA USA

**Keywords:** Paratransgenesis, Termite, Tn7 transposon, GFP, Gut, Bacteria

## Abstract

**Background:**

The Formosan subterranean termite, *Coptotermes formosanus* is an invasive urban pest in the Southeastern USA. Paratransgenesis using a microbe expressed lytic peptide that targets the termite gut protozoa is currently being developed for the control of Formosan subterranean termites. In this study, we evaluated *Trabulsiella odontotermitis*, a termite-specific bacterium, for its potential to serve as a ‘Trojan Horse’ for expression of gene products in termite colonies.

**Results:**

We engineered two strains of *T. odontotermitis*, one transformed with a constitutively expressed GFP plasmid and the other engineered at the chromosome with a Kanamycin resistant gene using a non- disruptive Tn7 transposon. Both strains were fed to termites from three different colonies.

Fluorescent microscopy confirmed that *T. odontotermitis* expressed GFP in the gut and formed a biofilm in the termite hindgut. However, GFP producing bacteria could not be isolated from the termite gut after 2 weeks. The feeding experiment with the chromosomally engineered strain demonstrated that *T. odontotermitis* was maintained in the termite gut for at least 21 days, irrespective of the termite colony. The bacteria persisted in two termite colonies for at least 36 days post feeding. The experiment also confirmed the horizontal transfer of *T. odontotermitis* amongst nest mates.

**Conclusion:**

Overall, we conclude that *T. odontotermitis* can serve as a ‘Trojan Horse’ for spreading gene products in termite colonies. This study provided proof of concept and laid the foundation for the future development of genetically engineered termite gut bacteria for paratransgenesis based termite control.

**Electronic supplementary material:**

The online version of this article (doi:10.1186/s12866-016-0822-4) contains supplementary material, which is available to authorized users.

## Background

Termites are eusocial insects displaying division of labor, overlapping generations, and cooperative brood care [1]. Termites depend on cellulose as their food source and play an important role in the natural ecosystem by carbon recycling [2, 3]. However, in the urban environment certain termite species are considered serious pests [4]. The Formosan subterranean termite (FST), *Coptotermes formosanus*, is an invasive urban pest from China and is estimated to cause an economic loss of $1 billion annually in the US [5]. This termite species forms large underground colonies with tunnels and galleries; and, in a mature colony, the number of individual termites can reach more than a million [6, 7].

Chemical insecticides are widely used for termite control but are known to affect other non-target organisms [8]. Conventional biological control remains unsuccessful for termite control due the termites’ hygienic behavior, such as grooming, removal of diseased individuals, and incorporation of antimicrobial substances into nest material, in addition to immune responses [9]. Paratransgenesis, a technique involving genetically engineered symbionts as ‘Trojan Horses’ can bypass a termite’s various defense systems and is suggested as an alternative, chemical free method for termite control [9]. In termite colonies, workers forage, digest the food, and feed the rest of the colony via stomodeal and proctodeal food exchange known as trophallaxis [1]. This social behavior aids the spread of the “Trojan Horse” in the colony and makes termites a good model for paratransgenesis.

Workers of the FST harbor a complex and diversified microbial community of bacteria, protozoa, and archaea in their guts [10, 11]. FSTs have an obligate symbiotic relationship with three species of gut protozoa, namely *Pseudotrichonympha grassi*, *Holomastigotoides hartmanni*, and *Spirotrichonympha leidyi* [12]. These gut protozoa assist the termite workers with the digestion of cellulose and are essential for the survival of the termite colony [13]. A targeted anti-protozoal peptide consisting of a ligand with affinity to protozoa, fused to the lytic peptide Hecate has been shown to kill the gut protozoa [14]. In a previous study, genetically engineered yeast (*Kluyveromyces lactis)* expressing this ligand-Hecate fusion peptide was successfully used to kill termites by eliminating their gut protozoa [15]. Although the yeast, which is not a natural gut symbiont, provided proof for the ‘Trojan Horse’ concept, a termite specific bacterium would be uniquely adapted to the gut environment and thus be more likely to survive for prolonged periods in the gut and less likely to cause environmental contamination. A carefully designed paratransgenesis approach utilizing genetically engineered termite specific bacteria expressing an effector molecule that impacts the vitality of a termite colony directly (by killing termites) or indirectly (by killing obligate symbionts) could be developed as an alternative to conventional termite control or as a synergistic method in integrated pest management.

In a previous study, genetically engineered *Enterobacter cloacae* expressing an insecticidal toxin from *Photorhabdus luminescens* was shown to kill termites in lab experiments [16]. *Enterobacter cloacae* is frequently found in the termite gut and genetically engineered strains have been shown to be effectively introduced into termite colonies and survive long enough to express foreign gene product and be transferred amongst nest mates [17]. However, *Enterobacter cloacae* is not termite specific and can be pathogenic in nature [18].

*Trabulsiella odontotermitis* is a termite specific bacterium which was first isolated and described from the gut of the fungus growing termite *Odontotermes formosanus* from southern Taiwan [19]. A recent study showed that *T. odontotermitis* is frequently present in various species of fungus growing termites [20]. Genome sequencing of *T. odontotermitis* has shown many adaptations, such as the ability to switch between aerobic and anaerobic metabolism, increased capacity for bacterial competition and possible aflatoxin degradation ability, suggesting that it is an important facultative symbiont of termites [20]. In a comparative study between bacterial flora of introduced and native FST populations using 16S rRNA gene sequencing, strains related to *T. odontotermitis* were found in FSTs from China [11]. In addition, *T. odontotermitis* was isolated from the gut of the FST from Japan as one of the uricolytic bacteria [21]. In our previous study, we isolated *T. odontotermitis* from the gut of the FST from Louisiana, USA, and found that *T. odontotermitis* is fifty times more tolerant to ligand-Hecate than the concentration required to kill the gut protozoa [22]. With the ultimate goal in mind to engineer *T. odontotermitis* in the future to express ligand-Hecate for termite control, we tested if genetically engineered *T. odontotermitis* was able to survive and express foreign proteins in the termite gut, and be transferred among nest mates via trophallaxis (transfer of digestive fluids).

## Methods

### Plasmid construction

DNA encoding ELGFP6.1, a variant of GFP [23] was amplified from plasmid pTrcHis2-ELGFP6.1 –TOPO using primers GFP6.1_KpnI_Fw 5’TTATGGTACCGATCATGAGTAAAGGAGAACTTTTC3’ containing a *Kpn*I restriction site and a start codon and GFP6.1_XhoI_Rv 5’TTGACTCGAGATCATTTGTATAGTTCATCC3’ with *Xho*I restriction site and a stop codon (restriction sites underlined). The product was digested with *Kpn*I and *Xho*I restriction enzymes and was ligated in frame with the Shine-Dalgarno sequence into plasmid pSF RecA Delta LexA constitutive (Product name- pSF-OXB20, Product Code: OG50, Oxford Genetics, UK) also digested with *Kpn*I and *Xho*I. The new plasmid was designated as pCT-ELGFP 6.1. Correct orientation of the insert was confirmed by PCR and sequencing using primers OGP-F2 5’TGTCGATCCTACCATCCA 3’and OGP-R2 5’AGTCAGTCAGTGCAGGAG 3’. Plasmid pCT-ELGFP 6.1 was maintained in *E.coli* DH5 alpha cells.

### Confirmation of the *attTn7*site in the *Trabulsiella odontotermitis* chromosome

*Trabulsiella odontotermitis* AS-7737 was isolated from the FST gut in a previous study [22]. To confirm presence of the *attTn7* site in the *T. odontotermitis* chromosome, *glmS* and *pstS* genes of *E.coli* MG1655, *Citrobacter koseri* ATCC BAA-895, *Salmonella enterica* subsp*. enterica* serovar Typhimurium LT2, *Klebsiella pneumoniae* subsp. *pneumoniae* HS1128 and *Enterobacter cloacae* EcWSU1 were aligned using ClustalX2 [24]. Two degenerate primers GLMS_CT_Fw and PSTS_CT_Rv were designed from the conserved regions of *glmS* and *pstS* genes, respectively (Additional file 1: Figure S1). The primers were presumed to amplify the C-terminus coding region of *glmS* gene, the inter-genic region between *glmS* and *pstS* and the N-terminus coding region of the *pstS* gene. Genomic DNA of *T. odontotermitis* was extracted using the DNeasy Blood & Tissue Kit (Qiagen 69504) and was subjected to PCR using primers GLMS_CT_Fw and PSTS_CT_Rv. The amplified product was cloned in pCR®2.1-TOPO® (Invitrogen K4660-01) according to manufacturer’s instructions and was subsequently sequenced at Macrogen, MD, USA. The sequence obtained was used to confirm the presence of *attTn7* site by comparing it with the consensus *attTn7* site as described previously [25]. At the time of the experiment the whole genome sequence of *T. odontotermitis* was not yet published. However, we were able to confirm the sequence obtained from this experiment by comparing it to *T. odontotermitis glmS* and *pstsS* genes obtained from the *T. odontotermitis* whole genome project made available to us by James Estevez, University of Puget Sound (Personal communication).

### Preparation of electrocompetent cells and transformation of *Trabulsiella odontotermitis*

*Trabulsiella odontotermitis* culture was grown to OD of 0.6 and 1 ml of the culture was centrifuged at 10,000 g at 4 °C. The cell pellet was washed two times with 1 ml ice cold sterile distilled water followed by two washes with 1 ml ice cold 10 % glycerol solution. The cells were then suspended in 50 μl of 10 % glycerol and mixed with 50 ng of pCT-ELGFP 6.1 for electroporation in a 2 mm gap electroporation cuvette (Eppendorf electroporator 2510 at 2.5 kV). For transposition, cells were co-transformed with 100 ng each of PUC18R6KT-mini-Tn7T-Km and pTNS-3 (provided by Dr. Herbert Schweizer, Colorado State University) using the same electroporation conditions. PUC18R6KT-mini-Tn7T-Km is a plasmid with a Tn7 transposon containing a *KanR* cassette flanked by a FRT site within Tn7L and Tn7R sequences [26]. pTNS-3 is a helper plasmid expressing *tnsABCD* [27]. After electroporation, cells were grown in 1 ml SOC medium for 1–2 h and were spread on LB+ Kanamycin 50 μg/ml (LB+ Kan 50) plates in different ten fold dilutions. Plates were incubated at 37 °C for 24 h and Kanamycin resistant colonies were selected for further analysis. Plates were observed under a UV trans-illuminator (UVP) and *T. odontotermitis* transformed with pCT-ELGFP 6.1 was detected by the presence of fluorescent colonies. Cells from individual colonies were also observed under a fluorescent microscope (Leica DM RXA2 fluorescent microscope, 100x oil, N.A = 1.3, excitation 480 nm and emission 508 nm). For cells transformed with pUC18R6KT-mini-Tn7T-Km and pTNS-3, 100 Kanamycin resistant colonies were re-streaked on LB+ Kan 50 plates and were subsequently stored as glycerol stocks at −80 °C until further analysis. To check the utility of pCT-ELGFP 6.1 to express GFP in other wild type bacteria, *Klebsiella* sp. AMC81C9, *Enterobacter cloacae* CMC61A1, *Enterobacter aerogenes* MCE84A10 and *Citrobacter koseri* E710D3 (all isolated previously from the termite gut) were also transformed [22].

### Confirmation of insertion of *KanR* cassette at *attTn7*site

Genomic DNA was isolated from five Kanamycin resistant isolates transformed with pUC18R6KT-mini-Tn7T-Km and pTNS-3 from the previous step using DNeasy Blood & Tissue Kit (Qiagen 69504). The DNA from these isolates along with the DNA from wild type *T. odontotermitis* was used for PCR using GLMS_CT_Fw and PSTS_CT_Rv. PCR products were run on 1 % agarose gel. Approximately 700 bp of the PCR product were sequenced from each end using GLMS_CT_Fw and PSTS_CT_Rv primers at Macrogen, MD, USA.

### Termite collection

Workers and soldiers of the Formosan subterranean termite (FST); *Coptotermes formosanus* were collected from three different colonies in New Orleans, LA using untreated inground bait stations. Colonies were designated as Colony A (collected from Canal Street, on 10/29/2013), Colony B (collected from Joe Brown Park 10/28/2013) and Colony C (collected from Little Woods, on 10/28/2013). Termites were brought back to the lab in plastic containers containing moist filter paper.

### Feeding experiment

Feeding experiments were carried out with two different strains of *T. odontotermitis, T. odontotermitis-*pGFP and *T. odontotermitis*-Km^r^:: *Tn7*. Strains *T. odontotermitis-*pGFP and *T. odontotermitis*-Km^r^:: *Tn7* were grown to OD 0.6 in LB+ Kan 50 broth. Cells in 1 ml volume were pelleted down and washed three times with equal volume of sterile water. The cells were suspended in 500 μl of sterile water and were added to cellulose discs prepared as previously described [15].

For the feeding experiment with *T. odontotermitis*-pGFP, groups of 200 worker termites and 20 soldier termites were collected from each of three colonies (A, B and C) and were fed on cellulose discs containing *T. odontotermitis*-pGFP for two days in a petri dish. All the experiments including the controls consisted of three replicates from each colony. After two days, guts of five randomly collected workers were dissected, pooled and homogenized in sterile saline solution (0.85 % W/V NaCl). The homogenate was serially diluted and was spread on LB+ Kan 50 plates. The plates were incubated at 37 °C for 24 h and fluorescent colonies were observed and counted under UV light (FirstLight® UV Illuminator, UVP). The numbers of bacteria per termite gut were estimated by dividing the bacterial colony count by five. After confirmation of bacterial intake in all the replicates, on day 3, termites were moved to a new petri dish containing a sterile cellulose disc moistened with sterile tap water. Every other day, five worker termites from each plate were dissected for bacterial isolation as described above. The experiment was carried out until no more fluorescent colonies were observed on LB+ Kan 50 plates (after 18 days). For the first 4 days, after the termites were moved to a new petri dish, three worker guts from each plate were dissected and observed under the fluorescent microscope (Leica DM RXA2 fluorescent microscope).

For the feeding experiment with *T. odontotermitis*-Km^r^ :: *Tn7*, 400 termite workers and 40 termite soldiers from each colony were fed on cellulose discs containing *T. odontotermitis*-Km^r^ :: *Tn7*. After two days, five worker termites were randomly selected and were used for isolation of Kanamycin resistant bacteria as described above. On the third day, 200 termite workers and 20 termite soldiers were moved to a new petri dish containing a sterile cellulose disc as soon as presence of Kanamycin resistant bacteria was confirmed in all the samples. Every two or three days, five worker termites from each petri dish were used to isolate and enumerate Kanamycin resistant bacteria.

### Bacterial horizontal transfer

For the bacterial transfer experiment, 200 termite workers and 20 termite soldiers from each colony were fed for two days on a cellulose disc containing 1 % Sudan red G (91282 Fluka), which stains the fat body of the termites red [28]. These termites were designated as recipient termites (no prior exposure to *T. odontotermitis*-Km^r^ :: *Tn7*). Termites fed on *T. odontotermitis*-Km^r^ :: *Tn7* were designated as donor termites. On the third day post feeding, the uptake of *T. odontotermitis*-Km^r^ :: *Tn7* was confirmed in donors and they were mixed with the recipient termites in the ratio of 1:1 and 1:25 (Additional file 2: Figure S2). After every two days, five recipient worker termites were randomly selected and were dissected for isolation of Kanamycin resistant bacteria as described above. The experiment was carried out for 2 weeks until recipient termites were indistinguishable from the donors due to the fading of the fat body stain. Two types of negative controls were used in the experiment; the first control contained 200 termite workers and 20 termite soldiers that were fed on cellulose containing non-engineered wild type *T. odontotermitis* and the second control consisted of 200 worker termites and 20 soldier termites that were fed on moistened sterile cellulose discs. The controls were treated in the same way as described for the experiments involving *T. odontotermitis*-Km^r^ :: *Tn7* and *T. odontotermitis*-pGFP.

A total of 96 randomly selected isolates from the feeding and transfer experiments were subjected to PCR and 700 bp of the PCR product were sequenced from each end with primers GLMS_CT_Fw and GLMS_CT_Fw to confirm the isolates were in fact *T. odontotermitis*-Km^r^ :: *Tn7*. No Kanamycin resistant bacteria could be isolated from any of the controls during the course of the experiment. PCR and sequencing of all the 96 isolates collected during the experiment confirmed that all tested the isolates were *T. odontotermitis*-Km^r^ :: *Tn7* .

### Consumption and mortality analysis

All of the cellulose discs were weighed before the start of the feeding experiment for each of the four treatments (control, with no added bacteria, *T. odontotermitis* wild type, *T. odontotermitis*-pGFP, *T. odontotermitis*-Km^r^ :: *Tn7*, or with Sudan red). At the end of the feeding experiment, cellulose discs were dried and weighed again to measure the consumption. Termite mortality in each replicate was calculated by counting the live termite workers at the end of the experiment.

### Statistical analysis

All statistical analysis was done using SAS 9.3 (SAS Institute, Cary, NC). PROC UNIVARIATE was used to check the data for normality. PROC MIXED with SLICE function was used to analyze the data from the feeding experiment from all days and all the replicates. PROC MIXED was used to analyze the data for consumption. PROC LOGISTIC adjusted with Tukey’s test was used to calculate probabilities of termite mortality for various treatments.

## Results and discussion

### Transformation with a constitutively expressed plasmid leads to strong but transient GFP expression in termite gut bacteria

In a previous study we transformed *Trabulsiella odontotermitis* with a lactose/ IPTG inducible GFP plasmid [22]. We were able to retrieve engineered *T. odontotermitis* via culture from the termite gut thereby confirming that the strain was ingested by the termites; however, we were not able to visually detect GFP expression in the termite gut [22]. Failure to induce the promoter due to insufficient lactose concentration was the most likely cause for the lack of expression. Our previous experiments also showed that with a low copy number plasmid, it is difficult to observe GFP expression against the termite gut’s auto-fluorescence (unpublished data). To overcome these issues, we constructed a new high copy number plasmid (pCT-ELGFP 6.1) in this study, which has a variant of GFP under the control of a strong constitutively expressed promoter RecA ∆LexA and *KanR* gene.

Transformation of *T. odontotermitis* with pCT-ELGFP6.1 conferred Kanamycin resistance. Transformed colonies showed fluorescent phenotype when observed under UV light. Even single cells from transformed colonies showed bright fluorescence when observed under a fluorescent microscope (Additional file 3: Figure S3), confirming the strong constitutive expression of GFP provided by this multicopy plasmid. *Trabulsiella odontotermitis* harboring pCT-ELGFP 6.1 was designated as *T. odontotermitis-*pGFP. Three other bacteria species isolated from the termite gut (*Klebsiella* sp. AMC81C9, *Enterobacter cloacae* CMC61A1, *Enterobacter aerogenes* MCE84A10) also showed strong constitutive expression of GFP after being transformed with pCT-ELGFP6.1, which suggests that the plasmid can be used to tag a variety of wild type bacteria. The results suggest that a construct with RecA ∆LexA promoter can be utilized in our future goal of engineering *T. odontotermitis* to express ligand-Hecate.

After the termites were fed for two days on *T. odontotermitis-*pGFP, fluorescent Kanamycin resistant colonies were successfully isolated from the gut homogenate of workers from all three termite colonies. The rapid uptake of *T. odontotermitis*-pGFP is consistent with the previous studies showing immediate presence of engineered bacteria and yeast in the termite gut, sometimes within hours after being added to the termite diet [15, 17, 29].

**Fig. 1:**
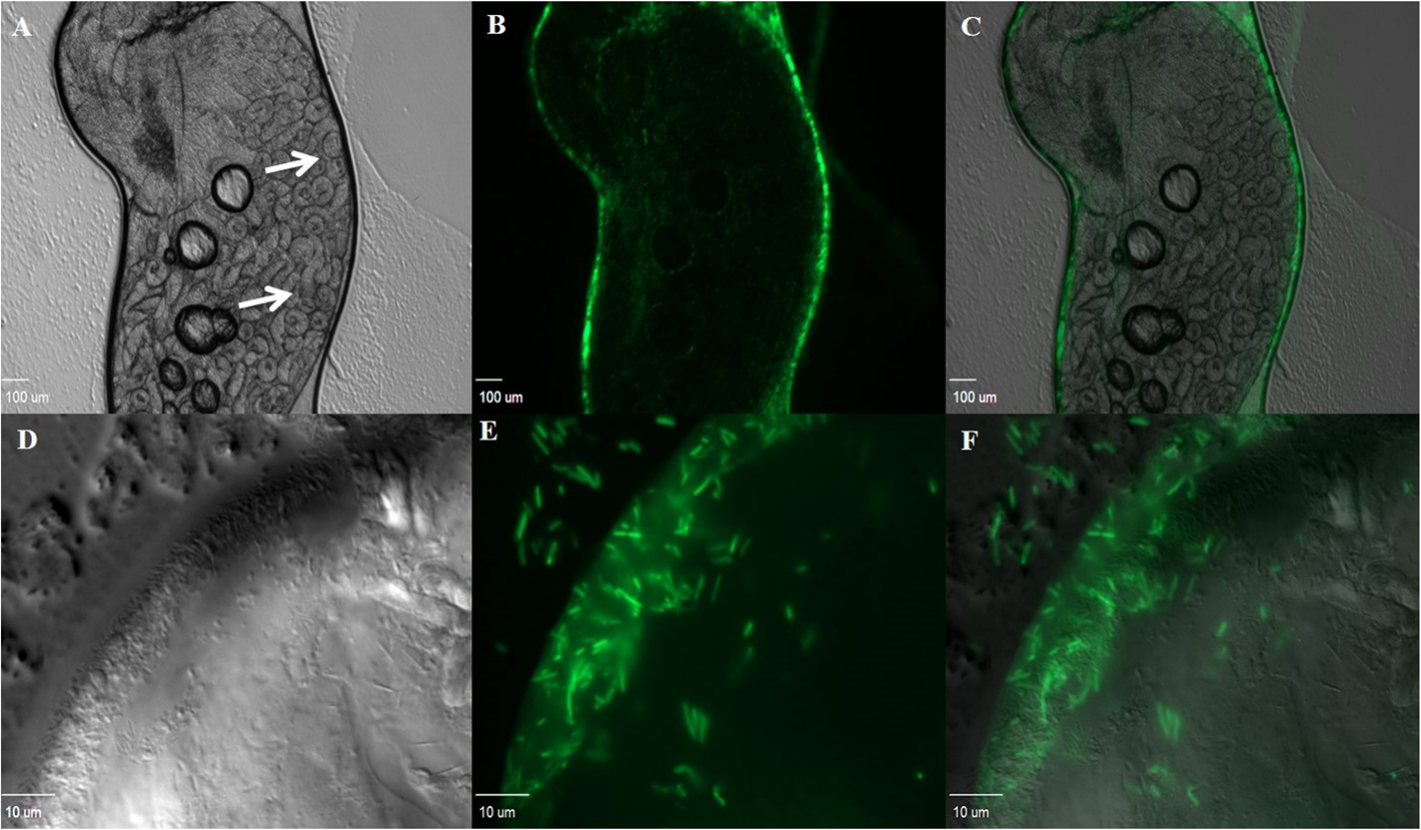
Termite hindgut observed under a Leica DM RXA2 fluorescent microscope after feeding on diet containing *T. odontotermitis*-pGFP. **a** 5× Differential interference contrast (DIC), *white arrows* pointing at termite gut protozoa. **b** 5× fluorescent, *T. odontotermitis*-pGFP seen concentrated at the hindgut wall. **c** Overlay of A and B, *T. odontotermitis*-pGFP seen in the close vicinity of gut protozoa. **d** 100× DIC, magnified image of the termite hindgut wall. **e** 100× fluorescent, magnified image of the termite hindgut wall showing *T. odontotermitis*-pGFP cells expressing GFP. **f** Overlay of **d** and **e**

Expression of GFP by *T. odontotermitis-*pGFP in the gut was directly observed via fluorescent microscopy. The *T. odontotermitis-*pGFP was concentrated in the hindgut region. In most instances, *T. odontotermitis-*pGFP appeared to have formed a biofilm around the hindgut paunch region, which contains the gut protozoa (Fig. 1a–f). Colonization of *T. odontotermitis* of the largely anaerobic hindgut region of termite workers suggests a preference for a niche with low oxygen levels in the gut [30]. Similar results were observed in case of fungus growing termites, where *T. odontotermitis* was predominately found in the hindgut paunch region [20]. A recent genome sequencing and gene expression study has shown that *T. odontotermitis* can switch between aerobic and anaerobic lifestyle [20]. The ability of *T. odontotermitis* to colonize the vicinity of the protozoa in the termite gut is an important attribute for a successful paratransgenesis system to achieve termite control via killing the cellulose-digesting protozoa [14, 15]. Colonization in the hindgut region would aid in the direct delivery of the protozoacidal peptide (ligand-Hecate) to the gut protozoa and would prevent the digestion of expressed ligand-Hecate by protease enzymes found in the termite midgut [31].

**Fig. 2:**
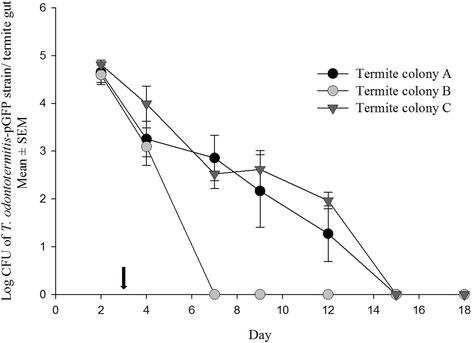
Number of *T. odontotermitis*-pGFP cells recovered from the gut of the termites of three different colonies after feeding for 2 days on cellulose discs containing *T. odontotermitis*-pGFP. The *arrow* indicates the day when the termites were moved to a sterile diet. The experiment had three replicates for each colony and 200 worker and 20 soldier termites were used for each replicate. Error bars indicate Standard Error of Mean (SEM)

During the first two days of feeding on cellulose discs containing *T. odontotermitis-*pGFP, the number of *T. odontotermitis-*pGFP cells that could be isolated on Kanamycin media ranged from 3.96 to 6.49 × 10^4^ per termite gut (Fig. 2) and, no significant differences were found in the bacterial counts from all three colonies (*P* = 0.7696, PROC MIXED with SLICE, Additional file 4: Table S1). After two days termites were switched to a diet of sterile cellulose discs and the number of *T. odontotermitis-*pGFP cells isolated from the termite gut rapidly decreased. By day 7, no more Kanamycin resistant bacteria could be isolated from the termites of colony C and by day 12 the number of *T. odontotermitis-*pGFP cells in guts of termites from colonies A and B also dropped below a detectable threshold (Fig. 2). Throughout the experiment, no Kanamycin resistant bacteria could be isolated from the guts of the control termites.

Even though the use of pCT-ELGFP6.1 to transform *T. odontotermitis* improved expression in the termite gut compared to a previously used plasmid with a lactose/ IPTG inducible promoter [22], it is not suitable to study long term survival of engineered bacteria in the termite gut and transfer among nest mates because GFP expression was lost too quickly. Loss of expression was most likely due to the loss of the plasmid by the bacteria in the absence of selective antibiotic pressure [32]. Since the experiment was carried out in the laboratory, it is currently not known how fast and by what mechanisms plasmids might be lost in field colonies. However, the loss of the marker in the lab experiments prompted us to construct *T. odontotermitis*-Km^r^ :: *Tn7*, a strain engineered to express *KanR* from the chromosome, to hopefully provide more stable expression.

### *Trabulsiella odontotermitis* engineered at chromosomal level at the *attTn7* site

When engineering any wild bacterial strain with the goal of preserving its functionality, care needs to be taken not to disrupt any of its vital genes. The use of Tn5 and Mu transposons involves random transposition events [33, 34] that can disrupt important genes required for efficient performance in the natural environment.A site specific Tn7 transposon, however, inserts in the bacterial chromosome without disrupting any of the host genes [35]. In most bacteria, the Tn7 transposon recognizes the *attTn7* site present within the C terminus region of a highly conserved glucosamine synthetase (*glmS*) gene [25]. Tn7 insertions take place 25 bp after the coding region without gene disruption [25, 35]. These features make Tn7 transposon an ideal tool for tagging wild type bacteria without any prior knowledge about the genome.

To successfully utilize a Tn7 transposon system, presence of *attTn7* in the chromosome at a neutral location is desired. A primer set GLMS_CT_Fw and PSTS_CT_Rv was designed with the goal to amplify a putative *attTn7* site present at the C-terminus coding region of the *glmS* gene. A PCR product with approximately 500 bp was obtained using primers GLMS_CT_Fw and PSTS_CT_Rv. Comparison of the sequenced PCR product to the sequences present in the NCBI GenBank database confirmed that this product contained the C-terminus coding region of the *glmS* gene, the inter-genic region between *glmS* and *pstS* and the N-terminus region of the *pstS* gene. Comparison of the sequence to a consensus *attTn7* sequence also revealed the presence of an *attTn7* site at the C-terminus region of *glmS* gene [25]. No known gene or *Tn7* transposon was detected in the inter-genic region between the *glmS* and *pstS* genes. The sequence was further confirmed by comparing it with the whole genome sequence of *T. odontotermitis* [20]. The presence of the attTn7 site confirmed using the primers GLMS_CT_Fw and PSTS_CT_Rv further corroborates its universal existence.

**Fig. 3:**
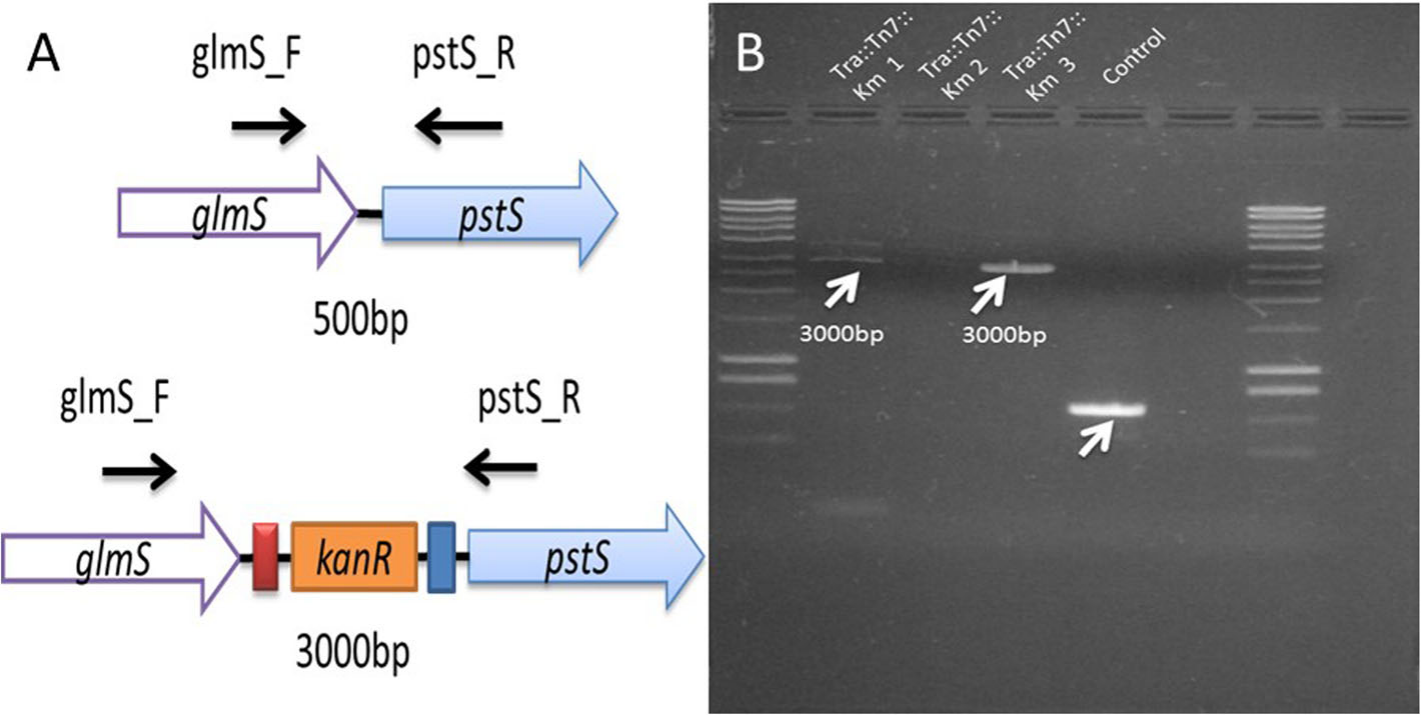
**a** Integration of *kanR* gene in the chromosome of *T. odontotermitis* using a *Tn7* transposon integration; glmS_F and pstS_R show the position and direction of primers used to confirm the integration **b** PCR based confirmation of integration of *kanR* gene in the *T. odontotermitis* chromosome using glmS_F and pstS_R primers. Tra:: Tn7:: km 1,2,3 are the three different isolates after a Tn7 transposition, control is the wild type *T. odontotermitis*

PCR amplification of the DNA of three isolates co-transformed with pUC18R6KT-mini-Tn7T-Km and pTNS-3 using primers GLMS_CT_Fw and PSTS_CT_Rv to confirm the insertion of *KanR* cassette in the *T. odontotermitis* chromosome resulted in a PCR product of ~3000 bp. Amplification using control wild type *T. odontotermitis* resulted in a PCR product of ~500 bp (Fig. 3a, b). Partial sequencing of 3000 bp PCR product confirmed the correct orientation of the inserted *KanR* cassette. *Trabulsiella odontotermitis* containing a *KanR* cassette in the chromosome was designated as *T. odontotermitis*-Km^r^ :: *Tn7*. The successful insertion of *KanR* cassette in the intergenic region between *glmS* and *pstS* proved its usefulness in a non-disruptive chromosomal tagging. This approach will be utilized in the future to insert a ligand-Hecate gene in the *T. odontotermitis* chromosome without disrupting any of its native genes. This is the first report of genetic manipulation in the genus *Trabulsiella* at the chromosome level.

### Chromosomally engineered *T. odontotermitis* is maintained in the termite gut for 3 weeks after ingestion

**Fig. 4:**
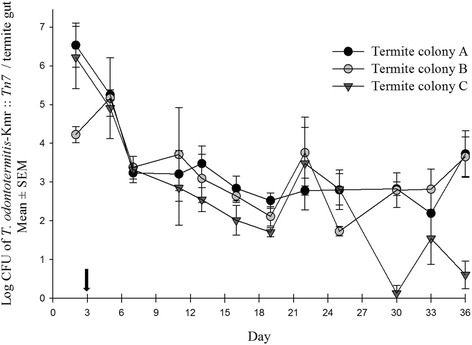
Number of Kanamycin resistant *T. odontotermitis*-Km^r^ :: *Tn7* recovered from the gut of the termites of three different colonies. The arrow on the X-axis indicates the day when termites were moved to a sterile diet. The experiment had three replicates for each colony and 200 worker and 20 soldier termites were used for each replicate. Error bars indicate Standard Error of Mean (SEM)

Similar to the results showing a rapid intake of *T. odontotermitis*-pGFP strain by termites, *T. odontotermitis*-Km^r^ :: *Tn7* was also isolated from the gut of workers from all three colonies within two days of feeding. It is likely that bacteria were ingested within hours as shown previously [17, 29]. Only at the beginning of the experiment (at day 2 of feeding), there was significant difference in the bacterial count among colonies (*P* = 0.0349, PROC MIXED with SLICE, Additional file 4: Table S2), with termites from Colony B having less Kanamycin resistant bacteria compared to Colony A and C (Fig. 4). However, once the termites were moved to sterile cellulose discs, no significant differences were found in the bacterial counts from all three colonies until day 22 (PROC MIXED with SLICE, Additional file 4: Table S2). The bacterial count sharply decreased in all three colonies until day 6 (3 days after the diet was switched to a sterile cellulose disc). From day 6 to day 22, the bacteria count oscillated between 10^3^ and 10^4^ bacteria/termite gut in all three colonies. After day 22, the bacterial counts from colony C decreased steadily, however, in the other two colonies (A, B) the engineered bacteria strain persisted and even at the end of the experiment (day 36) an average of 4.9×10^3^ bacteria cells per termite gut were still detected in both the colonies.

Our results show that *T. odontotermitis* is maintained in the gut for at least 3 weeks irrespective of the colony. This should be more than enough time for a future *T. odontotermitis* ‘Trojan Horse” that will be engineered to express lytic peptides to spread throughout a termite colony and kill the vital gut protozoa in workers. Previous studies have shown that 1 μM of ligand-Hecate can kill all the gut protozoa within 10 min in vitro [14]. Injection of 0.3 μL of 500 μM ligand-Hecate solution killed all three species of gut protozoa within 24 h. Ingested genetically engineered *K. lactis* expressing ligand-Hecate also killed all the gut protozoa within 3 weeks. After the loss of gut protozoa termites die within 2 weeks [14, 15].

In this study the load of genetically engineered *T. odontotermitis* per termite gut was counted which is different from the previous studies where the main focus was on number of the termites ingesting the bacteria [16, 17, 29]. In case of genetically engineered *K. lactis*, the number of yeast cells isolated from the termite gut after 3 weeks were approximately ten times higher than that of *T. odontotermitis* [15]. However, in that study termites were continuously feeding on a diet containing the engineered yeast [15]. During this study, termites were allowed to feed on the cellulose diet containing *T. odontotermitis*-Km^r^ :: *Tn7* for only two days. The results show that even without continuous replenishing of engineered bacteria through feeding, the bacteria are maintained in the gut at moderate levels (10^3^–10^4^ bacteria/ termite gut). This is a useful attribute for the future application of paratransgenesis where we intend to use a bait system to feed engineered bacteria to termites. Only a fraction of the termite workers forages at any point in time and foraging sites may change. Thus, continuous feeding on the bait cannot be assured and the success of paratransgenesis depends on fast and efficient uptake, and survival of engineered bacteria in the termite gut, in addition to efficient spread to colony members that did not directly feed on the bait.

### Chromosomally engineered *T. odontotermitis* is horizontally transferred amongst nest mates

Horizontal transfer of termiticides is required in order to achieve a colony level elimination and has been established with many termiticides [36, 37]. Previous studies have shown that termites can horizontally transfer bacteria and yeast via trophallaxis [15, 17, 29]. Horizontal spread of engineered *T. odontotermitis* throughout the colony was experimentally modelled by combining donors (termites that ingested *T. odontotermitis*-Km^r^ :: *Tn7*) and recipients in two ratios 1:1 and 1:25.

In the transfer experiment with the donor: recipient ratio 1:25, Kanamycin resistant bacteria could be isolated from the gut of the recipient termites from two of the three colonies after 2 days of mixing donor and recipient termites. There was no significant difference between the bacterial counts of the three termite colonies on day 4, 10 and 13 (PROC MIXED with SLICE, Additional file 4: Table S3). However, on day 2 and 7 the bacterial counts of the three termite colonies were significantly different from each other (PROC MIXED with SLICE, Additional file 4: Table S3). On day 16, no bacteria were recovered from any of the replicates from colony C and overall there was no significant difference between the bacterial counts from all the three termite colonies (*P* = 0.3991, PROC MIXED with SLICE, Additional file 4: Table S3). There were large differences in the bacterial counts among individuals even within the same colony (Additional file 5: Figure S4).

**Fig. 5:**
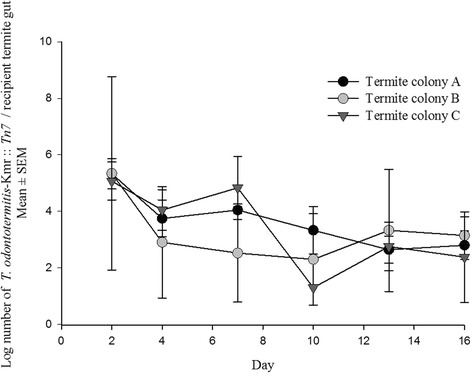
*T. odontotermitis*-Km^r^ :: *Tn7* recovered from the gut of the recipient termites (donor: recipient ratio 1:1) of three different colonies. The experiment had three replicates for each colony. Error bars indicate Standard Error of Mean (SEM)

In the transfer experiment with the donor: recipient ratio 1:1, Kanamycin resistant bacteria were isolated from the gut of the recipient termites from all three colonies just 2 days after combining donor and recipient termites. Except for day 7 (*P* = 0.04, PROC MIXED with SLICE, Additional file 4: Table S4), there was no significant difference in the bacterial counts in the guts of recipients from the three termite colonies for 16 days (PROC MIXED with SLICE, Additional file 4: Table S4). After day 16 the experiment was discontinued because there was no clear distinction between the donor and recipient termites due to the loss of coloration in the fat body of the recipients. At day 16, the average number of bacteria per recipient termite gut was around 7.5×10^3^ (Fig. 5).

In order to be successful in a bait system, *T. odontotermitis* must be horizontally transferable to the nest mates. These results suggest that *T. odontotermitis* has a better transfer efficiency amongst the nest mates than *K. lactis*. After 2 weeks the number of *T. odontotermitis* recovered from the termite gut was approximately five times higher in comparison to *K. lactis* [15]. A higher transfer efficiency of *T. odontotermitis* will aid in its spread throughout the termite colony which again is an important asset for the success of paratransgenesis.

### Consumption and mortality

For future application of *T. odontotermitis* as a ‘Trojan Horse’ for termite control in a bait system, non-repellency is an important aspect to ensure ingestion of a lethal dose [38]. Analysis of cellulose consumption suggested that addition of *T. odontotermitis* did not deter termites from feeding. The type of treatment, i.e. whether termites were fed with either strain of the genetically engineered bacteria (*T. odontotermitis*-pGFP or *T. odontotermitis*-Km^r^ :: *Tn7*) did not have any effect on the consumption of cellulose (*P* = 0.38,PROC MIXED). However, each colony reacted differently to different substrates and colony membership had a significant effect on the consumption (*P* = 0.004, PROC MIXED). In this study, there was no correlation between the probability of mortality and the type of treatment, suggesting that addition of *T. odontotermitis* to the diet does not result in increased mortality of termites (PROC LOGISTIC, Additional file 6: Figure S5).

## Conclusion

The study proved that 1. A termite specific bacterium, *T. odontotermitis* can be engineered with a plasmid and at chromosome level using a non-disruptive *Tn7* transposon based method to express foreign proteins in the termite gut. 2. Engineered *T. odontotermitis* was ingested by the termite and survived in the gut for at least 21 days. 3. Engineered *T. odontotermitis* is horizontally transferred amongst nest mates via social interactions. 4. *T. odontotermitis* does not have effect on termite mortality and diet consumption. This study provided proof of concept for the future development of genetically engineered termite gut bacteria for paratransgenesis based termite control. In the future we intend to genetically engineer *T. odontotermitis* to express ligand-Hecate using a constitutive promoter and a Tn7 transposition. Ultimately, engineered *T. odontotermitis* expressing ligand-Hecate will be used in bait and will be assessed as a termite control agent.

## Additional files

Additional file 1: Figure S1. Multiple alignment of *glmS* (top) and *pstS* (bottom) genes of *E.coli* MG1655, *Citrobacter koseri* ATCC BAA-895, *Salmonella enterica* subsp. enterica serovar Typhimurium LT2, *Klebsiella pneumoniae* subsp. pneumoniae HS1128 and *Enterobacter cloacae* EcWSU1. Frames show the region used for designing primers GLMS_CT_Fw and PSTS_CT_Rv respectively. (JPG 194 kb) Additional file 2: Figure S2. Termites feeding on a cellulose disc in bacterial transfer experiment; the white termites are the donor termites previously fed on cellulose diet with *T. odontotermitis*-Km^r^ :: *Tn7*, the pink termites are the recipient termites fed on cellulose diet with Sudan red, Donor: Recipient 1:1. (JPG 251 kb) Additional file 3: Figure S3.
*T. odontotermitis* transformed with pCT-ELGFP 6.1, observed under Leica DM RXA2 fluorescent microscope, 100x oil, N.A = 1.3, excitation 480 nm and emission 508 nm. (JPG 32 kb) Additional file 4: Supplementary tables. (XLSX 14 kb) Additional file 5: Figure S4.
*T. odontotermitis*-Km^r^ :: *Tn7* recovered from the gut of the recipient termites (donor: recipient ratio 1:25) of three different colonies. The experiment had three replicates for each colony. Error bars indicate Standard Error of Mean (SEM). (JPG 55 kb) Additional file 6: Figure S5. Probabilities of mortality of three termite colonies fed on cellulose diet with the addition of *T. odontotermitis*-Km^r^ :: *Tn7*, *T. odontotermitis* wild type, *T. odontotermitis*-pGFP, and Sudan red. The negative control consisted of cellulose only. There was no significant difference amongst the probabilities of mortality based on the type of diet consumed (*P* = 0.21, PROC LOGISTIC). (JPG 49 kb)
