# Supplementary figures and images for: Assessment of genetically engineered Trabulsiella odontotermitis as a ‘Trojan Horse’ for paratransgenesis in termites
Source: BMC Microbiol. 2016 Sep 5;16(1):202. doi: 10.1186/s12866-016-0822-4 (PMC5011783; doi:10.1186/s12866-016-0822-4)

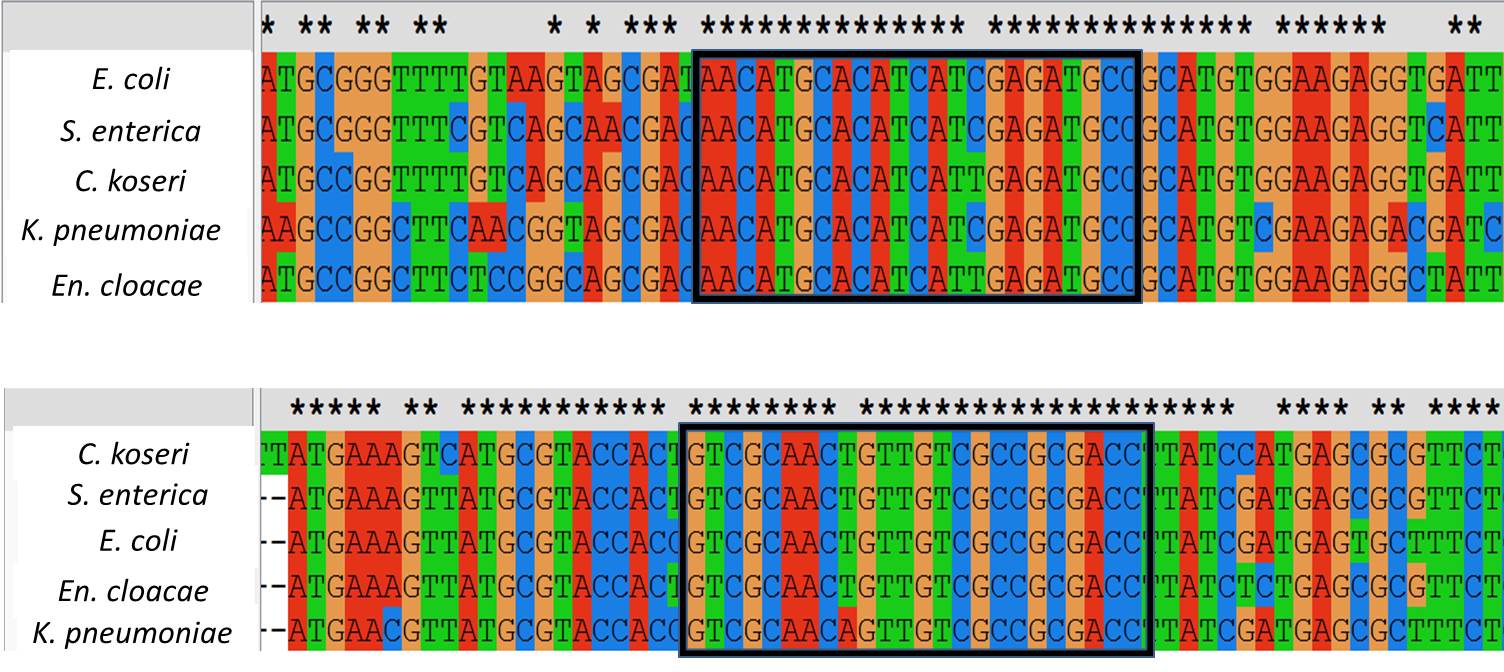

Supplement: Additional file 1: Figure S1. — Multiple alignment of glmS (top) and pstS (bottom) genes of E.coli MG1655, Citrobacter koseri ATCC BAA-895, Salmonella enterica subsp. enterica serovar Typhimurium LT2, Klebsiella pneumoniae subsp. pneumoniae HS1128 and Enterobacter cloacae EcWSU1. Frames show the region used for designing primers GLMS_CT_Fw and PSTS_CT_Rv respectively. (JPG 194 kb) [file 12866_2016_822_MOESM1_ESM.jpg]

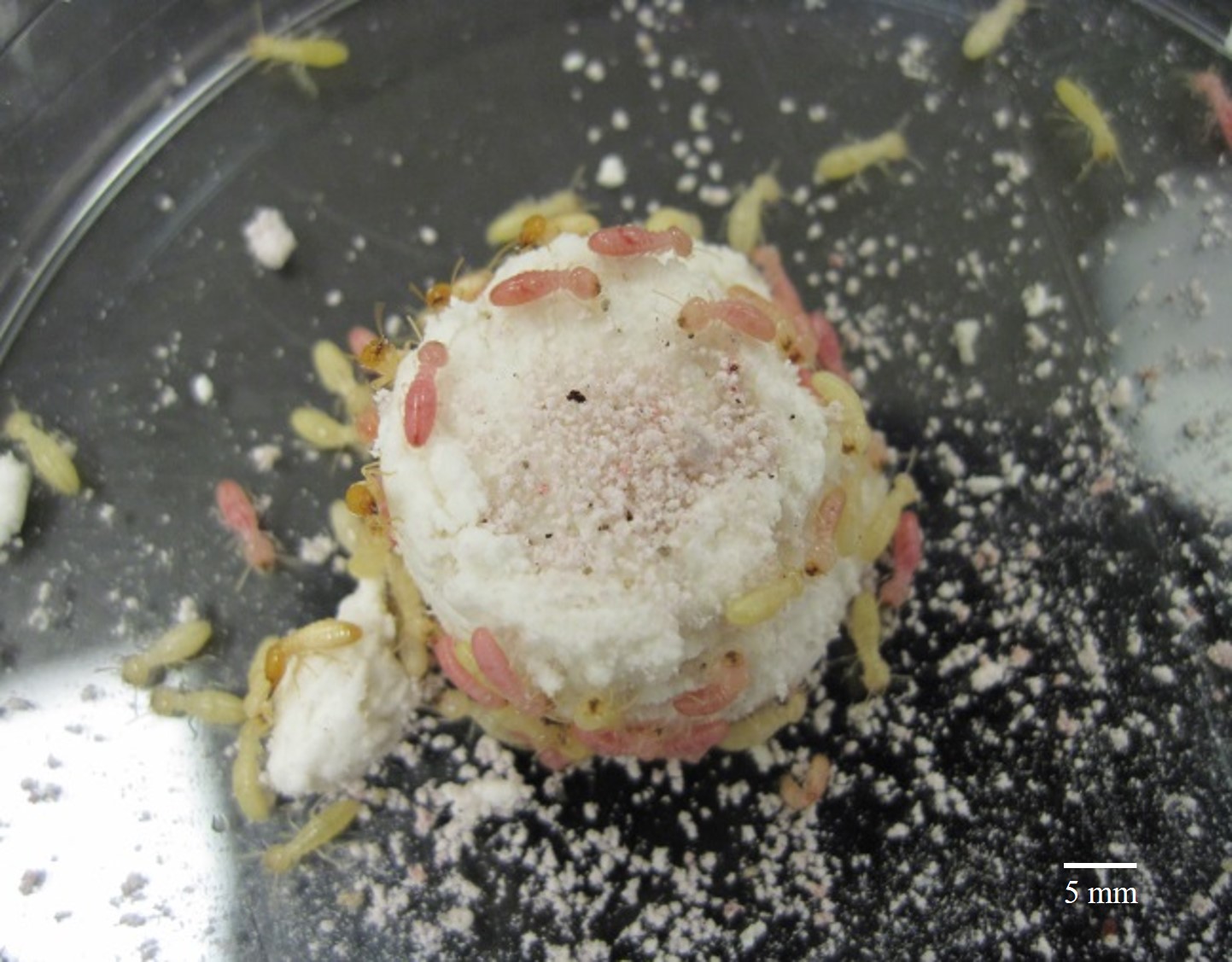

Supplement: Additional file 2: Figure S2. — Termites feeding on a cellulose disc in bacterial transfer experiment; the white termites are the donor termites previously fed on cellulose diet with T. odontotermitis-Kmr :: Tn7, the pink termites are the recipient termites fed on cellulose diet with Sudan red, Donor: Recipient 1:1. (JPG 251 kb) [file 12866_2016_822_MOESM2_ESM.jpg]

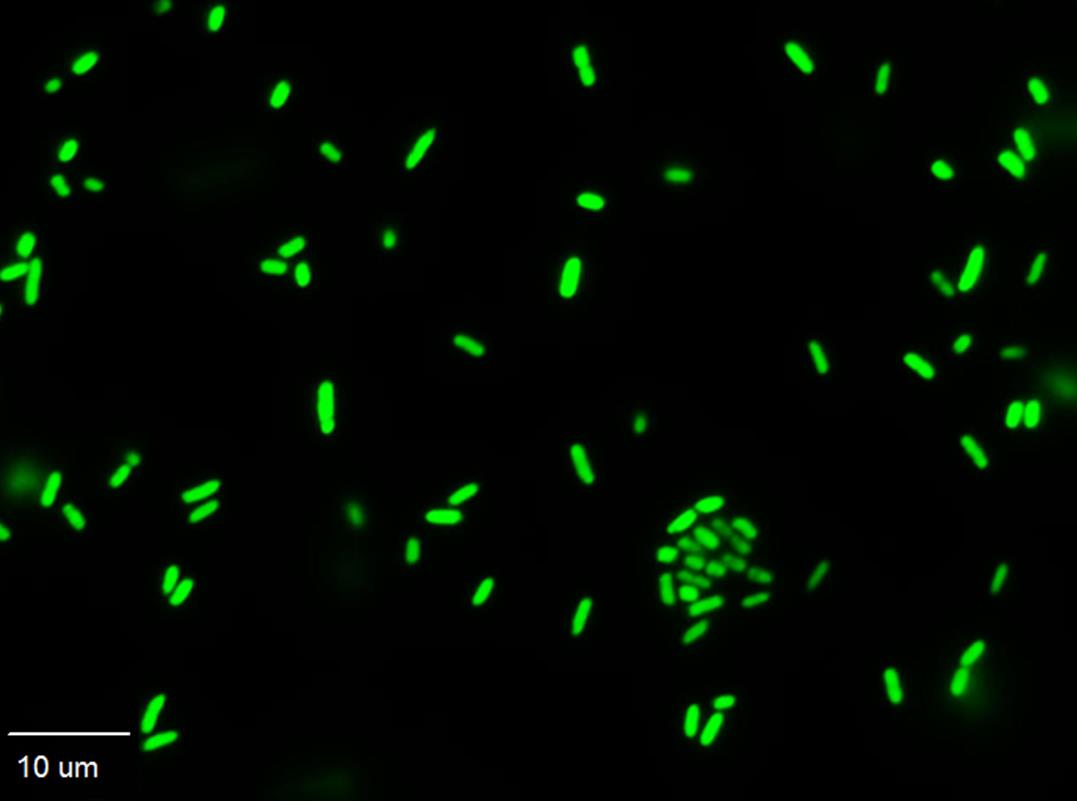

Supplement: Additional file 3: Figure S3. — T. odontotermitis transformed with pCT-ELGFP 6.1, observed under Leica DM RXA2 fluorescent microscope, 100x oil, N.A = 1.3, excitation 480 nm and emission 508 nm. (JPG 32 kb) [file 12866_2016_822_MOESM3_ESM.jpg]

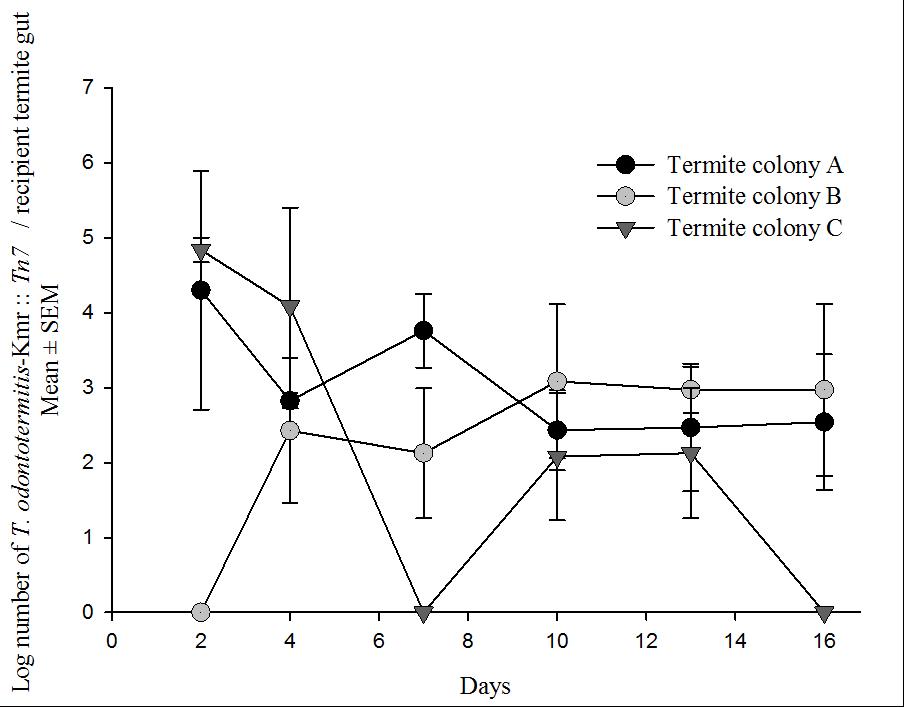

Supplement: Additional file 5: Figure S4. — T. odontotermitis-Kmr :: Tn7 recovered from the gut of the recipient termites (donor: recipient ratio 1:25) of three different colonies. The experiment had three replicates for each colony. Error bars indicate Standard Error of Mean (SEM). (JPG 55 kb) [file 12866_2016_822_MOESM5_ESM.jpg]

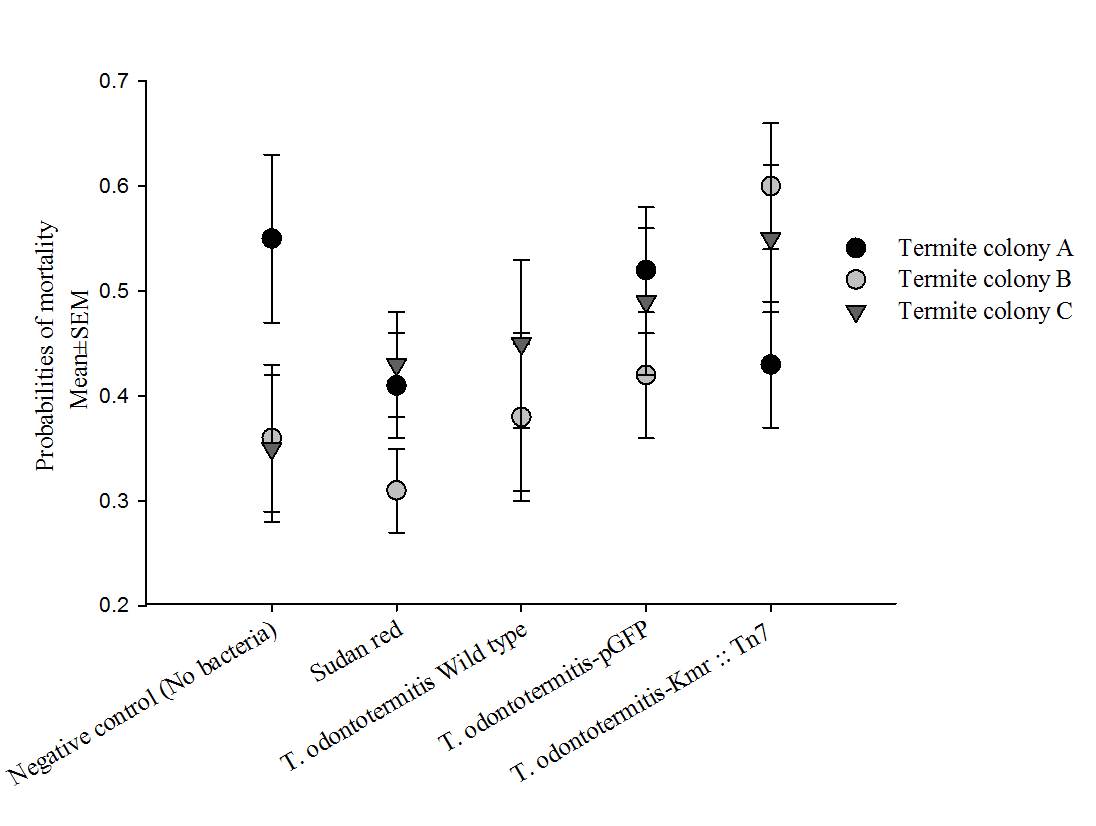

Supplement: Additional file 6: Figure S5. — Probabilities of mortality of three termite colonies fed on cellulose diet with the addition of T. odontotermitis-Kmr :: Tn7, T. odontotermitis wild type, T. odontotermitis-pGFP, and Sudan red. The negative control consisted of cellulose only. There was no significant difference amongst the probabilities of mortality based on the type of diet consumed (P = 0.21, PROC LOGISTIC). (JPG 49 kb) [file 12866_2016_822_MOESM6_ESM.jpg]
